# Clinical outcomes of hyperprogression based on volumetry in non‐small cell lung cancer after immune checkpoint inhibitor treatment

**DOI:** 10.1111/1759-7714.14539

**Published:** 2022-07-03

**Authors:** Jehun Kim, Taeyun Kim, Tae Won Jang, Hee Kang, Mi Hyun Kim, Seong Hoon Yoon, Choon‐Hee Son, Hyun‐Kyung Lee, Hyun‐Kuk Kim, Shin Yup Lee, Kyeong Choel Shin, Ji‐Yeon Han, Eun‐Ju Kang

**Affiliations:** ^1^ Division of Pulmonology, Department of Internal Medicine Kosin University College of Medicine, Kosin University Gospel Hospital Busan South Korea; ^2^ Department of Internal Medicine The Armed Forces Goyang Hospital Goyang‐si South Korea; ^3^ Department of Radiology Kosin University College of Medicine, Kosin University Gospel Hospital Busan South Korea; ^4^ Division of Pulmonology, Department of Internal Medicine Pusan National University Hospital Pusan South Korea; ^5^ Division of Pulmonology, Department of Internal Medicine Pusan National University Yangsan Hospital Yangsan South Korea; ^6^ Division of Pulmonology, Department of Internal Medicine Gwanghye General Hospital Pusan South Korea; ^7^ Division of Pulmonology, Department of Internal Medicine Inje University Busan Paik Hospital Busan South Korea; ^8^ Division of Pulmonology, Department of Internal Medicine Inje University Haeundae Paik Hospital Busan South Korea; ^9^ Division of Pulmonology, Department of Internal Medicine Kyungpook National University Chilgok Hospital Daegu South Korea; ^10^ Division of Pulmonology, Department of Internal Medicine Yeungnam University Hospital Daegu South Korea; ^11^ Department of Radiology Inje University Busan Paik Hospital Busan South Korea; ^12^ Department of Radiology Dong‐A University College of Medicine Busan South Korea

**Keywords:** hyperprogression, immune checkpoint inhibitor, non‐small cell lung cancer, volumetry

## Abstract

**Background:**

Hyperprogressive disease (HPD) is a novel pattern of the treatment course after immune checkpoint inhibitor (ICI) therapy in patients with non‐small cell lung cancer (NSCLC). This study aimed to investigate the clinical characteristics, outcomes, and associated factors of HPD using a semiautomatic volume measurement.

**Methods:**

This retrospective study enrolled patients with recurrent and/or metastatic NSCLC treated with ICIs between January 2015 and August 2019 at eight tertiary centers in Korea. HPD was defined according to the tumor growth kinetics and time to treatment failure. Tumor volume was measured using a semiautomatic software.

**Results:**

A total of 219 NSCLC patients with 35 HPD by volumetric measurement (HPDv) (15.9%) were enrolled. The median duration of overall survival (OS) and OS after ICI treatment (ICI‐OS) were 34.5 and 18.4 months, respectively. HPDv patients had significantly worse progression‐free survival (PFS) than progressive disease patients without HPDv (1.16 vs. 1.82 months, *p*‐value <0.001). ICI‐OS did not significantly differ between patients with HPDv and those without HPDv (2.66 vs. 5.4 months, *p* = 0.105). PD‐L1 expression lower than 50%, more than three metastatic sites, neutrophil‐to‐lymphocyte ratio equal to or higher than 3.3, and hemoglobin level lower than 10 were found to be associated with HPDv.

**Conclusions:**

There is no standardized definition of HPD. However, defining HPD in NSCLC patients treated with ICI using a semiautomatic volume measurement software is feasible.

## Graphical abstract

Our study evaluated hyperprogression in non‐small cell lung cancer patients treated with immune‐checkpoint inhibitor using a semiautomatic volume measurement. Patients experiencing hyperprogression by volumetric measurement had significantly worse progression‐free survival and overall survival after the initiation of immune‐checkpoint inhibitor than patients without it.

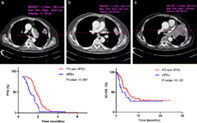

## INTRODUCTION

Since the introduction of immune checkpoint inhibitors (ICIs) targeting the programmed cell death protein 1 (PD‐1) and its ligand (PD‐L1) pathway, the treatment landscape for patients with lung cancer has dramatically changed. PD‐1 is an immune checkpoint receptor that is expressed in activated immune cells such as T cells, B cells, macrophages, and natural killer cells, while PD‐L1 is overexpressed in tumor cells and promotes immune escape.^1^ Due to their special characteristics, tumor response to ICIs could present an atypical pattern compared with the tumor response to cytotoxic chemotherapies. For example, pseudoprogression, an unconventional response pattern resulting from an intratumoral infiltration of immune cells,^2^ and hyperprogressive disease (HPD), an unexpected radiological tumor growth, have been linked to ICI treatment.

There has been increasing interest in this pattern of non‐small cell lung cancer (NSCLC). A study reported an incidence rate of 13.8% for HPD in patients with NSCLC receiving ICI treatment.^3^ This value in a Western group is similar to that reported in an Asian population, with a rate of 14.3%.^4^ NSCLC patients with HPD have poorer survival outcomes than those without HPD.^3^, ^4^ Several clinical markers, such as older age,^5^ female sex,^6^ PD‐L1 status,^7^ and a high neutrophil‐to‐lymphocyte ratio (NLR),^4^ have been suggested to reflect worse clinical outcomes. As the usage of ICI increases, it is plausible that the incidence of HPD will increase further. This may provoke additional socioeconomic burden to any healthcare system, considering that the economic feasibility of ICI has not yet been established.^8^

Given the unexpected rapid progression of tumors in response to ICI treatment, HPD may relate to poor progression‐free survival (PFS) and overall survival (OS).^9^, ^10^ Various parameters such as tumor growth rate (TGR), tumor growth kinetics (TGK), and time to treatment failure (TTF) have been introduced to precisely define HPD,^4^, ^9^, ^11^ although a consistent definition has not been made. Moreover, considering the different characteristics of HPD, the traditional method for assessing tumor response, Response Evaluation Criteria in Solid Tumors (RECIST),^12^ which calls for the diameter of the tumor, might not fully explain the unique nature of HPD. For example, during rapidly emerging progression of NSCLC, HPD, could be overestimated when evaluated using RECIST.^13^ In this context, a recent study showed that volumetric measurement is better than one‐dimensional analysis (RECIST) in evaluating HPD in patients with NSCLC receiving ICI treatment.^4^ In terms of measuring tumor response after ICI treatment, the volumetric method may be more useful and accurately deliver clinical implications than the diametric method.^4^, ^14^

Therefore, the present study aimed to evaluate the clinical characteristics and outcomes of HPD in patients with NSCLC treated with ICI using semiautomatic volumetric measurements.

## METHODS

### Patients

**FIGURE 1:**
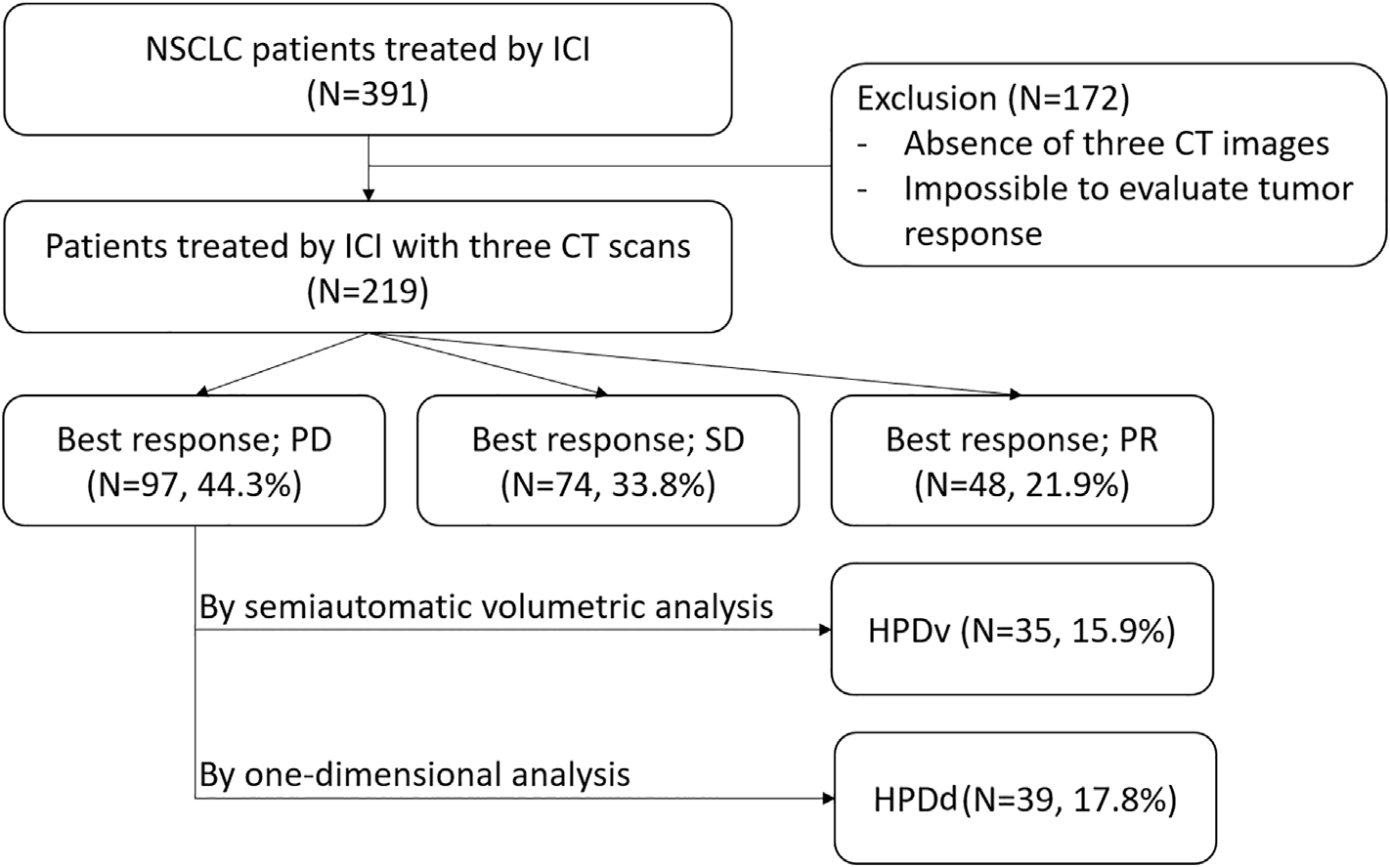
Flow chart of the patient selection process

In this retrospective study, we reviewed the electronic medical records of 391 patients diagnosed with advanced‐stage NSCLC treated with humanized, high‐affinity, selective PD‐1/PD‐L1 inhibitors (nivolumab, pembrolizumab, atezolizumab, and durvalumab) at eight tertiary referral centers in South Korea between January 2015 and August 2019. Of them, 172 patients with no available CT scans before or after the treatment and had no targetable lesions according to RECIST version 1.1^12^ were excluded. Finally, 219 patients were included in the final analysis (Figure 1).

The following data were collected: age, sex, smoking history, Eastern Cooperative Oncology Group performance status, previous history of surgery or radiation therapy, tumor pathology, PD‐L1 expression status, sites of metastasis, previous chemotherapy, type of ICI, best overall response to ICI, NLR (by dividing neutrophil count by lymphocyte count), platelet‐to‐lymphocyte ratio (PLR, calculated by dividing platelet count by lymphocyte count), hemoglobin (Hb) level, albumin level, and lactate dehydrogenase level.

This study was reviewed and approved by the Institutional Review Board of Kosin University Gospel Hospital (approval No. 2019–08‐001). The study was conducted following the Declaration of Helsinki. All procedures were performed in accordance with the relevant guidelines and regulations.

### Assessment of HPD

Three radiologists (H. Kang, J. Y. Han and E. J. Kang) reviewed three sequential CT images: baseline (within 1 month before ICI treatment), before baseline (within 1–2 months before baseline), and after baseline (1–2 months after ICI treatment). One target lesion was included, whereas nontarget and new metastatic lesions were excluded. The lesions were assessed on axial images using a mediastinal window (window level, 45 Hounsfield units [HU]; window width, 400 HU). HPD was measured using two different methods: HPD by diametric measurement using the longest diameter of the tumor (HPDd) and semi‐automated volumetric assessment (HPDv).

**FIGURE 2:**
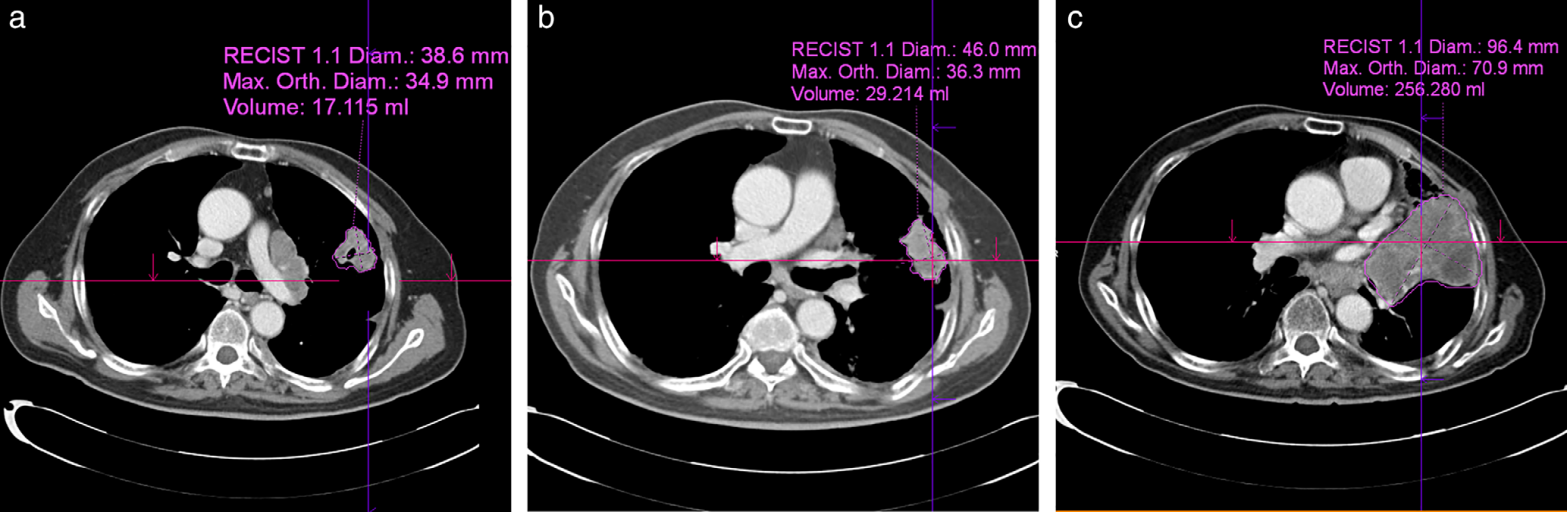
The longest diameter measurement and volumetric measurement performed using a semiautomatic software. (a) Before baseline. (b) Baseline. (c) After baseline. Diam, diameter; Max. Orth., maximum orthogonal.

Figure 2 shows three examples of these measurements. First, the longest diameter of the target lesion on the axial image was manually measured by a radiologist using a digital caliper based on RECIST version 1.1 (a, 38.6 mm; b, 46.0 mm; and c, 96.4 mm in Figure 2): D_base_, D_before_, and D_after_ indicate the tumor diameter at baseline, before baseline, and after ICI, respectively. The measured diameters of the target lesions were extrapolated to spherical volumes using the following formula: 4/3πr^3^, where r represents half the measured diameter of the tumor. This approach to measure tumor volume was used to estimate HPDd.

Volumetric measurements of the tumor were performed using a commercially available semiautomatic software package (syngo.via version VB30, Siemens Healthcare). Based on the longest diameter, the system automatically measured the orthogonal diameter of the tumor (a, 34.9 mm; b, 36.3 mm; and c, 70.9 mm in Figure 2). The system automatically then traces and renders the tumor border and measures the volume of the tumor (a, 17.115 ml; b, 29.214 ml; and c, 256.28 ml in Figure 2): V_base_, V_before_, and V_after_ indicate the tumor volume at baseline, before baseline, and after ICI, respectively. If the automatically drawn area included incorrect regions such as an adjacent normal organ, atelectasis, or pleural effusion, the radiologist corrected the outline.

Among several methods for defining HPD, we adopted the TGK method introduced by Kim et al.,^4^ which calculated the difference in tumor volume from three serial CT scans: T_base_, T_before_, and T_after_ represent the CT scan timing at baseline, before baseline, and after immunotherapy, respectively. TGK_pre_ was defined as the difference in the volume of target lesions per unit of time between baseline and before baseline images: TGK_pre_ = (V_base−_V_before_/T_base−_T_before_). Similarly, TGK_post_ was defined as the difference in the volume of target lesions per unit of time between after baseline and baseline: TGK_post_ = (V_after−_V_base_/T_after−_T_base_). The TGK ratio was defined as TGK_post_/TGK_pre_. HPD was defined as^1^ TTF less than 2 months and^2^ more than two‐fold increase in TGK.^4^

### Measurement of outcome

Complete response (CR), partial response (PR), and stable disease (SD) were defined based on RECIST version 1.1. Objective response rate (ORR) was defined as the percentage of patients who experienced CR or PR after ICI treatment, while disease control rate (DCR) was defined as the ratio of patients who experienced CR, PR, and SD after ICI treatment. PFS was used to measure the outcomes related to discontinuation of ICI treatment and defined as the period from the date of ICI initiation to the date of tumor progression or the date of death from any cause. OS was defined as the period from either the date of diagnosis or the start of cancer treatment to the date of death from any cause. OS after ICI treatment (ICI‐OS) was defined as the period from the date of ICI treatment initiation to the date of death from any cause. Patients who were still on treatment were censored at the time of data collection. In addition, TTF was introduced to define the period from the date of ICI treatment initiation to the date of study withdrawal due to patient refusal, adverse events, follow‐up loss, disease progression, or death.

### Statistical analysis

The patients were divided into two groups according to the status of HPD (HPDv or HPDd). The differences between the groups (HPD or non‐HPD) were analyzed using a chi‐square test or the Fisher's exact test for categorical variables and the Student's *t*‐test or Mann–Whitney *U* test for variables with non‐normal distribution. Before an analysis considering several covariables, we calculated the correlations of OS, ICI‐OS, and PFS with both TGK ratio by volumetric measurement (TGKv) and diametric measurement (TGKd). Survival analysis was evaluated using the Kaplan–Meier method, and the differences between the two groups were assessed using the log‐rank test. A logistic regression model was used to evaluate the odds ratio (OR) and 95% confidence interval (CI) of HPD and non‐HPD. Variables with a *p*‐value of <0.1 in the univariable model were included in the multivariate model. A *p* value of <0.05 was considered statistically significant. All statistical analyses were performed using SPSS version 25.0 (SPSS Inc.).

## RESULTS

The baseline characteristics of the study participants according to the status of HPDv are shown in Table 1 (HPDd in Table S1). A total of 149 patients were included in the non‐HPDv group, while 35 patients were included in the HPDv group. Patients with HPDv showed a higher number of metastatic organs, higher NLR and PLR, and lower Hb and albumin levels than those with non‐HPDv.

**TABLE 1:** Clinical characteristics according to hyperprogressive disease (HPD) by the volumetric measurement

	All patients (*n* = 219)	Non‐HPD (*n* = 184)	HPDv (*n* = 35)	*p*‐value
Mean age	65.0 ± 9.0	65.1 ± 9.2	64.3 ± 7.9	0.56
<60	63 (28.8%)	51 (27.7%)	12 (34.3%)	
≥60	156 (71.2%)	133 (72.3%)	23 (65.7%)	
Gender, (%)				0.27
Male	169 (77.2%)	145 (78.8%)	24 (68.6%)	
Female	50 (22.8%)	39 (21.2%)	11 (31.4%)	
Smoking history, (%)				0.81
Ever smoker	157 (71.7%)	133 (72.3%)	24 (68.6%)	
Never smoker	62 (28.3%)	51 (27.7%)	11 (31.4%)	
ECOG PS (%)				
≤1	182 (83.1%)	153 (83.2%)	29 (82.9%)	1.0
>2	37 (16.9%)	31 (16.8%)	6 (17.1%)	
Previous operative therapy, (%)				1.0
No	190 (86.8%)	160 (87.0%)	30 (85.7%)	
Yes	29 (13.2%)	24 (13.0%)	5 (14.3%)	
Previous radiation therapy, (%)				
No	133 (60.7%)	110 (59.8%)	23 (65.7%)	0.64
Yes	86 (39.3%)	74 (40.2%)	12 (34.3%)	
*EGFR* mutation, (%)				0.9
No	20 (9.1%)	167 (90.8%)	3 (8.6%)	
Yes	199 (90.9%)	17 (9.2%)	32 (91.4%)	
Tumor pathology (%)				1.0
Non SQC	77 (35.2%)	65 (35.3%)	12 (34.3%)	
SQC	142 (64.8%)	119 (64.7%)	23 (65.7%)	
PD‐L1 expression status (%)				0.1
<50%	68 (33.0%)	53 (30.5%)	15 (46.9%)	
≥50	138 (67.0%)	121 (69.5%)	17 (53.1%)	
Bone metastasis				0.47
No	146 (66.7%)	125 (67.9%)	21 (60.0%)	
Yes	73 (33.3%)	59 (32.1%)	14 (40.0%)	
Brain metastasis				0.96
No	179 (81.7%)	151 (82.1%)	28 (80.0%)	
Yes	40 (18.3%)	33 (17.9%)	7 (20.0%)	
Liver metastasis				0.7
No	195 (89.0%)	165 (89.7%)	30 (85.7%)	
Yes	24 (11.0%)	19 (10.3%)	5 (14.3%)	
Adrenal metastasis				0.5
No	203 (92.7%)	172 (93.5%)	31 (88.6%)	
Yes	16 (7.3%)	12 (6.5%)	4 (11.4%)	
Metastatic site				0.01
≤2	186 (84.9%)	163 (88.6%)	23 (65.7%)	
>2	33 (15.1%)	21 (11.4%)	12 (34.3%)	
Treatment lines before ICI				0.08
1 or 2	182 (83.1%)	157 (85.3%)	25 (71.4%)	
3 or more	37 (16.9%)	27 (14.7%)	10 (28.6%)	
Type of ICI				0.56
Nivolumab	121 (55.3%)	98 (53.3%)	23 (65.7%)	
Pembrolizumab	84 (38.4%)	74 (40.2%)	10 (28.6%)	
Atezolizumab	13 (5.9%)	11 (6.0%)	2 (5.7%)	
Durvalumab	1 (0.5%)	1 (0.5%)	0 (0.0%)	
*Mean NLR*	3.7 ± 3.0	3.6 ± 2.8	4.6 ± 3.6	0.01
<3.3	127 (58.0%)	114 (62.0%)	13 (37.1%)	
≥3.3	92 (42.0%)	70 (38.0%)	22 (62.9%)	
Mean PLR	196.8 ± 112.4	188.4 ± 103.4	240.1 ± 144.9	0.05
<214	141 (64.4%)	124 (67.4%)	17 (48.6%)	
≥214	78 (35.6%)	60 (32.6%)	18 (51.4%)	
Hemoglobin level, g/dl	11.6 ± 1.8	11.7 ± 1.7	10.8 ± 1.9	< 0.001
<10	45 (20.5%)	29 (15.8%)	16 (45.7%)	
≥10	174 (79.5%)	155 (84.2%)	19 (54.3%)	
Albumin level, g/dl	3.9 ± 0.5	3.9 ± 0.5	3.6 ± 0.5	0.02
<3.5	46 (21.2%)	33 (18.1%)	13 (37.1%)	
≥3.5	171 (78.8%)	149 (81.9%)	22 (62.9%)	
LDH, U/l	388.6 ± 255.4	386.8 ± 269.2	396.9 ± 180.4	0.99
<450	111 (72.1%)	91 (71.7%)	20 (74.1%)	
≥450	43 (27.9%)	36 (28.3%)	7 (25.9%)	

Abbreviations: ECOG PS, Eastern Cooperative Oncology Group performance status; HPDv, patients with HPD assessed by volumetric method; ICI, immune‐checkpoint inhibitors; NLR, neutrophil‐lymphocyte ratio; PLR, platelet‐lymphocyte ratio; SQC, squamous cell carcinoma.

The median duration of ICI in all study participants was 3.1 months (range, 1 day to 30.7 months). The ORR and DCR in all patients were 21.9% and 55.7%, respectively. The best overall responses were PR in 48 patients (21.9%), SD in 74 patients (33.8%), and PD in 97 patients (44.3%). Of all study participants, 35 (15.9%) were classified as having HPDv, while 39 (17.8%) were classified as having HPDd.

**FIGURE 3:**
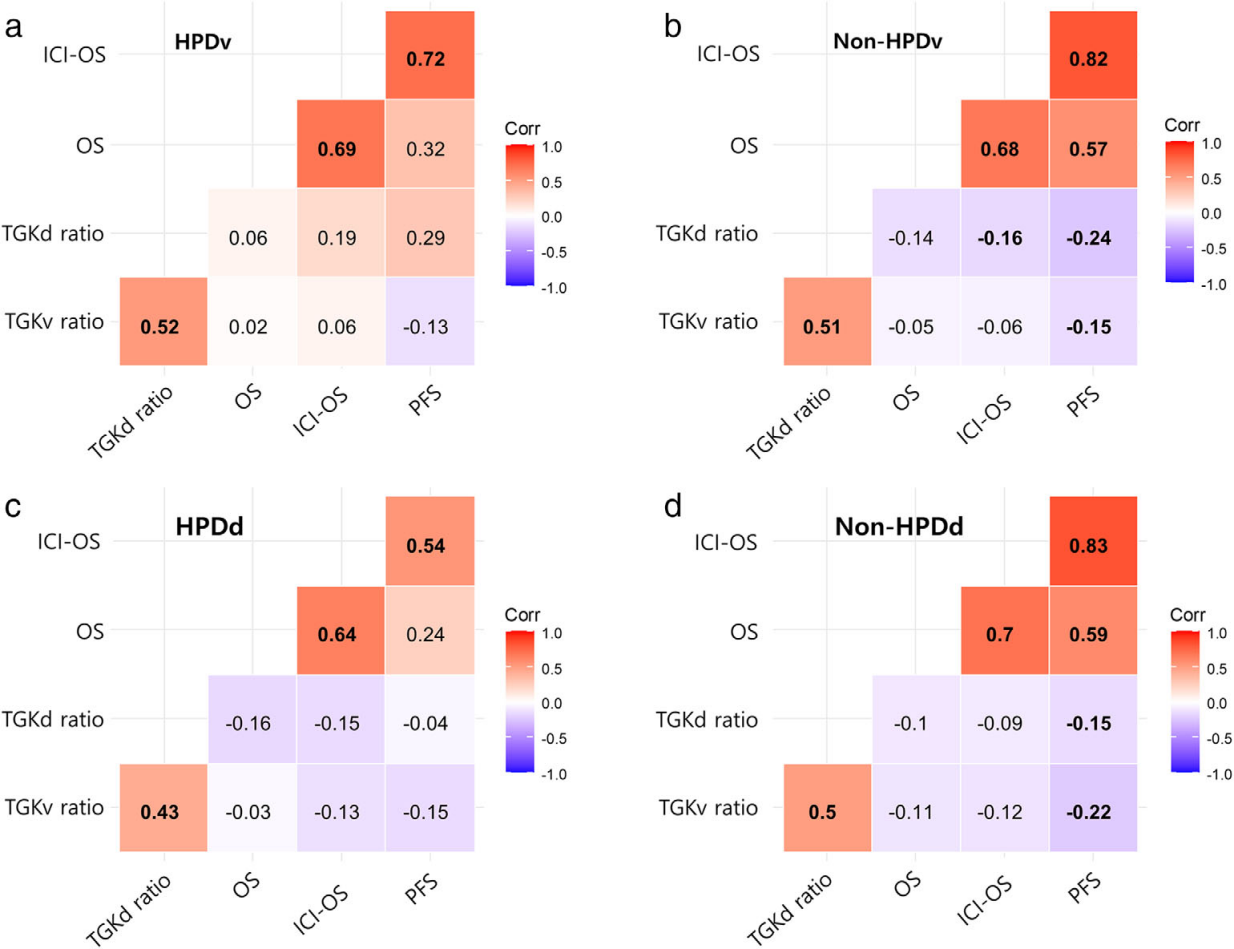
Spearman's rank correlation matrix. Values show the spearman rank results (significant correlations are in bold). Red areas represent significant negative correlations, blue areas represent significant positive correlation, and white areas represent nonsignificant correlations

The correlations according to the HPD status are shown in Figure 3. The significance levels and correlation coefficients varied between survival outcomes and TGK ratios. Positive correlations were observed between survival indicators (PFS, OS, and ICI‐OS) and TGK ratios. Negative correlations were observed between the TGK ratios and survival indicators.

**FIGURE 4:**
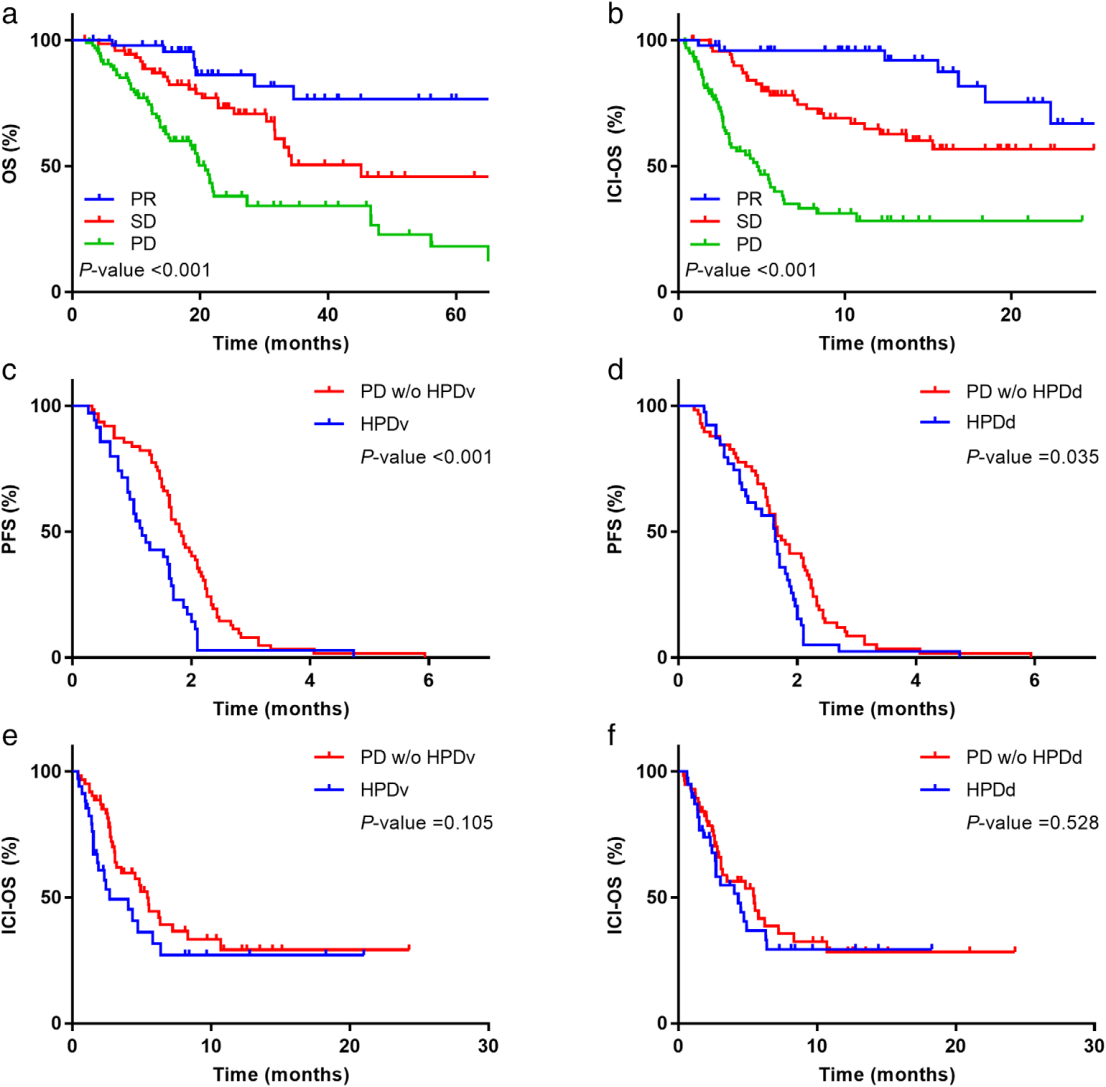
(a) OS and (b) ICI‐OS according to the response categories. Comparison of PFS between patients with HPD and those without HPD according to (c) HPDv and (d) HPDd status. Comparison of ICI‐OS between patients with HPD and those without HPD according to (e) HPDv and (f) HPDd status. OS, overall survival; ICI‐OS, overall survival, defined as the time from immunotherapy; HPD, hyperprogressive disease; HPDv, HPD defined by volumetry; HPDd, HPD defined by diameter.

The median OS in all participants was 34.5 months. A significant difference was observed in the OS between tumor responses (PR not reached, SD in 45.1 months, and PD in 20.8 months, *p* < 0.001) (Figure 4a). The median ICI‐OS in all participants was 18.4 months. Patients who presented with PR, SD, and PD had better outcomes (not reached, not reached, and 4.7 months, respectively, *p* < 0.001) (Figure 4b).

In terms of PFS, PD patients with HPDv were found to have poor outcomes than those without HPDv (1.16 vs. 1.82 months, *p* < 0.001, Figure 4c). Similarly, PD patients with HPDd had poor outcomes than those without HPDv (1.63 vs. 1.66 months, *p*‐value = 0.035, Figure 4d). However, no significant differences were observed in the ICI‐OS between PD patients with HPDv and those without HPDv (2.66 vs. 5.4 months, *p* = 0.105, Figure 4e) and between PD patients with HPDd and those without HPDd (4.33 vs. 5.46 months, *p* = 0.528, Figure 4f).

Table 2 presents the clinical factors affecting HPDv using univariable and multivariable logistic regression analyses. In the univariable analysis, PD‐L1 expression, number of metastatic sites, previous cancer treatment, NLR and PLR ratio, Hb level, and albumin level were found to be significant. In the multivariable model, PD‐L1 expression lower than 50%, more than three metastatic sites, NLR equal to or higher than 3.3, and Hb level lower than 10 were found to be associated with HPD.

**TABLE 2:** Factors affecting hyperprogression using univariable and multivariable logistic regression analyses

Parameters	Odds ratio	95% CI	*p*‐value
**Univariable**			
PD‐L1, <50% vs. ≥50%	2.014	0.937–4.332	0.073
Metastasis site, ≥3 vs. <3	4.05	1.761–9.314	0.001
Treatment lines before ICI, 3 or more vs. 1 or 2	2.326	1.005–5.384	0.049
NLR, ≥3.3 vs. <3.3	2.756	1.305–5.820	0.008
PLR, ≥214 vs. <214	2.188	1.053–4.545	0.036
Hemoglobin level, <10 vs. ≥10	4.501	2.075–9.763	<0.001
Albumin level, <3.5 vs. ≥3.5	2.668	1.220–5.835	0.014
**Multivariable**			
PD‐L1, <50% vs. ≥50%	2.447	1.051–5.696	0.038
Metastasis site, ≥3 vs. <3	2.759	1.030–7.392	0.044
NLR, ≥3.3 vs. <3.3	2.542	1.068–6.046	0.035
Hemoglobin level, <10 vs. ≥10	3.911	1.684–9.081	0.002

Abbreviations: ICI, immune‐checkpoint inhibitor; NLR, neutrophil‐lymphocyte ratio; PLR, platelet‐lymphocyte ratio.

## DISCUSSION

In this retrospective multicenter observational study, clinical characteristics and outcomes including ORR, DCR, PFS, OS, and ICI‐OS were evaluated, defining HPD based on the new semiautomated volumetric method and diametric method using the longest tumor diameter. High PD‐L1 expression rate, increased number of metastatic organ sites, high NLR, and low Hb level were associated with increased OR for predicting HPD. Patients presenting with HPDv showed poorer PFS than those without HPDv. Considering that there is no clear definition of HPD, only a few studies have measured HPD in Asian patients with NSCLC using an in‐house volume measurement software; moreover, relatively poor survival outcomes were observed in patients experiencing HPD. The results of this study may provide useful insights into the natural course of ICI therapy and warrant further studies to identify the predictive markers for HPD.

In the present study, both tumor kinetic models using semiautomatic volume measurement and TTF were used to define HPD. The application of tumor kinetics for identifying HPD has been adopted in several previous studies. For example, a study in patients with advanced‐stage head and neck cancer found that the presence of HPD defined by an increase in TGK of >2 was correlated with a shorter PFS, which is consistent with our findings.^11^ Another study measured TGK as the sum of the largest diameter of the target lesion before and after ICI therapy and defined HPD as the difference in the ratio exceeding 50%.^15^ In addition, Kim et al. compared volume‐based TGK to diameter‐based TGR and reported a high concordance rate of 99.6%.^4^ Our study showed a matching rate of 89% between volumetric and diametric measurements. The decreased concordance rate might have been due to the method used to evaluate the target lesion, considering only one prominent site with quick and easy comparison.

Because the term HPD implicates both massive changes in tumor size and rapid deterioration in a short period, TTF has been utilized in several studies to define hyperprogression,^3^, ^13^, ^16^, ^17^ showing a glimpse of evidence supporting its use in the evaluation of HPD. For example, a TTF of <2 months was additionally used to define and predict HPD, and patients satisfying this criterion presented poor survival outcomes.^4^, ^18^ Moreover, considering that at least three serial CT images are required to measure TGR and TGK, diagnosing HPD using these parameters might not always be feasible in real clinical practice. In this case, TTF may be informative.

In our data, HPDv and HPDd were identified in 15.9% and 17.8% of all patients receiving ICI, respectively. The incidence of HPD in previous studies ranged from 4% to 29%.^4^, ^9^, ^11^, ^19^ This inconsistency may have originated from an unestablished definition of HPD. In defining HPD, a precise measurement of changes in tumor size and TGR is important. We used the volume‐based TGK ratio using a commercially available semiautomatic volume measurement software package. The RECIST version 1.1 has been widely applied by clinicians to define tumor responses.^12^ However, as it is based on unidimensional diametric measurements, volumetric methods have been introduced to evaluate tumor response more precisely. Three‐dimensional volume measurement is likely to better reflect true tumor size growth,^20^ and the method yielded significantly lower intra‐ and interobserver variabilities.^21^

In our data, as in a previous study,^4^ there was a significant difference in HPDv‐stratified OS according to the presence of HPD, while there was no significant difference in HPDd‐stratified OS. Volumetric measurements may better reflect real clinical outcomes than diametric measurements. As the diametric measurement assumes that the shape of the tumor is spherical, an error may occur. Volumetric measurements reliably account for the real shape of the target lesion rather than assuming a perfectly spherical shape.^22^ The volumetric assessment of tumors is difficult and labor‐intensive. However, recent improvements in imaging software have allowed radiologists to perform semi‐automated assessment of tumor volume much more simply and easily. This method has been used in lung cancer screening research, showing superiority for both size and growth determination of lung nodules.^23^ Greenberg et al. also reported its applicability in evaluating the response to lung cancer treatment.^24^

Patients with HPDv or HPDd showed shorter PFS than those without HPD. This finding is in line with the results of previous studies.^9^, ^15^ In the group with recurrent and/or metastatic NSCLC who received ICI therapy, those with HPD had shorter PFS (hazard ratio: 4.7, 95% CI: 2.9–7.4) than those without HPD.^9^ Among patients treated with ICI, more than 40% of patients experienced PD as the best response to immunotherapy, and the median PFS was only 2.1 months. In patients treated with conventional chemotherapy, 30% of patients had PD as their best tumor response, and the median PFS was 3.9 months.^15^ Unlike PFS, a significant difference was not observed in the ICI‐OS. Abbar et al. defined HPD using TGR and TGK and reported that OS during HPD was not different between patients with and without this condition,^10^ which is consistent with the findings of another study.^13^

Multivariable analysis revealed that four clinical factors were significantly associated with an increase in OR in patients with HPD: low PD‐L1 expression rate, more than three metastatic organ sites, high NLR, and low Hb level. First, the number of metastatic sites may play an important role in the development of HPD. A previous study has shown that a high number of metastatic sites before ICI therapy is associated with HPD.^13^ Similarly, a recent meta‐analysis demonstrated that the number of metastatic sites is positively correlated with the risk of HPD.^25^ Second, a readily obtainable marker, NLR, might be associated with the development of HPD. Lymphocytes play a pivotal role in the action of anti‐PD‐1/PD‐L1 agents, and their activation and intratumor invasion are essential for ICI treatment. A previous study has shown that patients with high pre‐ICI NLR may experience inferior survival outcomes.^26^ In addition, a few studies have reported a potential predictive role of NLR in the course of NSCLC patients receiving ICIs.^27^, ^28^ Third, a low Hb level was correlated with an increased OR for HPD. Anemia is a hematological anomaly commonly observed in patients with lung cancer undergoing cancer treatment.^29^ A recent study has demonstrated that reduced baseline Hb level is an unfavorable prognostic marker in advanced NSCLC patients with ICI,^30^ while other studies in various parts have suggested that low Hb levels are associated with HPD in HCC patients with ICI.^31^, ^32^

Our study had several limitations. First, it was retrospective in nature; only patients with complete serial (baseline, before baseline, and after baseline) CT images were selected, which may have led to selection bias or misclassification. The results of a multicenter hospital‐based investigation in the current study may mitigate this limitation. Second, the median follow‐up period (defined as time from ICI to death or last follow‐up) was only 6.1 months, which is relatively short for evaluating the effects of ICI treatment on survival. However, given that the current study aimed to investigate HPD that appears shortly after treatment with ICI and the RECIST working group recommended at least 4 weeks to evaluate the tumor response after treatment,^33^ results from this study may provide useful insights to the outcomes of HPD. Third, our study included patients who had been initially diagnosed with advanced stage, as well as those with stages 1 and 2 who underwent curative surgery and stage 3 who received concurrent chemoradiation. Therefore, there was a relatively large difference between OS and ICI‐OS. In future studies, large‐scale research should be conducted in patients with recurrent lung cancer treated with ICIs.

In conclusion, in this study, we identified 15.1% of HPD patients with NSCLC treated with ICIs. HPD was measured using the semiautomatic volumetric method, which may be feasible in clinical practice. However, its generalizability and applicability may merit further consideration. PFS was shorter in patients with HPD than in those without HPD. Multivariable analysis showed that low PD‐L1 expression rate, high NLR, more than three metastatic sites, and low Hb levels were associated with the development of HPD. Future studies are needed to validate our results and develop practical criteria for identifying HPD after treatment with ICIs.

## CONFLICT OF INTEREST

The authors declare that they have no competing interests.

## Supporting information


**Table S1** Supporting Information

## References

[1] Topalian SL , Taube JM , Anders RA , Pardoll DM . Mechanism‐driven biomarkers to guide immune checkpoint blockade in cancer therapy. Nat Rev Cancer. 2016;16(5):275–87. 27079802 10.1038/nrc.2016.36 PMC5381938

[2] Rocha P , Hardy‐Werbin M , Naranjo D , Taus Á , Rodrigo M , Zuccarino F , et al. CD103+CD8+ lymphocytes characterize the immune infiltration in a case with Pseudoprogression in squamous NSCLC. J Thorac Oncol. 2018;13(10):e193–e6. 29775806 10.1016/j.jtho.2018.05.008

[3] Russo GL , Moro M , Sommariva M , Cancila V , Boeri M , Centonze G , et al. Antibody–fc/FcR interaction on macrophages as a mechanism for hyperprogressive disease in non–small cell lung cancer subsequent to PD‐1/PD‐L1 blockade. Clin Cancer Res. 2019;25(3):989–99. 30206165 10.1158/1078-0432.CCR-18-1390

[4] Kim Y , Kim CH , Lee HY , Lee S‐H , Kim HS , Lee S , et al. Comprehensive clinical and genetic characterization of hyperprogression based on volumetry in advanced non–small cell lung cancer treated with immune checkpoint inhibitor. J Thorac Oncol. 2019;14(9):1608–18. 31195179 10.1016/j.jtho.2019.05.033

[5] Champiat S , Dercle L , Ammari S , Massard C , Hollebecque A , Postel‐Vinay S , et al. Hyperprogressive disease is a new pattern of progression in cancer patients treated by anti‐PD‐1/PD‐L1. Clin Cancer Res. 2017;23(8):1920–8. 27827313 10.1158/1078-0432.CCR-16-1741

[6] Kanjanapan Y , Day D , Wang L , Al‐Sawaihey H , Abbas E , Namini A , et al. Hyperprogressive disease in early‐phase immunotherapy trials: clinical predictors and association with immune‐related toxicities. Cancer. 2019;125(8):1341–9. 30768786 10.1002/cncr.31999

[7] Kim JY , Lee KH , Kang J , Borcoman E , Saada‐Bouzid E , Kronbichler A , et al. Hyperprogressive disease during anti‐PD‐1 (PDCD1) / PD‐L1 (CD274) therapy: a systematic review and meta‐analysis. Cancers (Basel). 2019;11(11):1699. 10.3390/cancers11111699 PMC6896059 31683809

[8] Thungappa S , Ferri J , Caglevic C , Passiglia F , Raez L , Rolfo C . Immune checkpoint inhibitors in lung cancer: the holy grail has not yet been found…. ESMO Open. 2017;2(1):e000162. 28761734 10.1136/esmoopen-2017-000162 PMC5519814

[9] Kim C , Kim K , Pyo K‐H , Xin C‐F , Hong M , Ahn B‐C , et al. Hyperprogressive disease during PD‐1/PD‐L1 blockade in patients with non‐small‐cell lung cancer. Ann Oncol. 2019;30(7):1104–13. 30977778 10.1093/annonc/mdz123

[10] Abbar B , De Castelbajac V , Gougis P , Assoun S , Pluvy J , Tesmoingt C , et al. Definitions, outcomes, and management of hyperprogression in patients with non‐small‐cell lung cancer treated with immune checkpoint inhibitors. Lung Cancer. 2021;152:109–18. 33385736 10.1016/j.lungcan.2020.12.026

[11] Saâda‐Bouzid E , Defaucheux C , Karabajakian A , Coloma VP , Servois V , Paoletti X , et al. Hyperprogression during anti‐PD‐1/PD‐L1 therapy in patients with recurrent and/or metastatic head and neck squamous cell carcinoma. Ann Oncol. 2017;28(7):1605–11. 28419181 10.1093/annonc/mdx178

[12] Eisenhauer EA , Therasse P , Bogaerts J , Schwartz LH , Sargent D , Ford R , et al. New response evaluation criteria in solid tumours: revised RECIST guideline (version 1.1). Eur J Cancer. 2009;45(2):228–47. 19097774 10.1016/j.ejca.2008.10.026

[13] Matos I , Martin‐Liberal J , García‐Ruiz A , Hierro C , Ochoa de Olza M , Viaplana C , et al. Capturing hyperprogressive disease with immune‐checkpoint inhibitors using RECIST 1.1 criteria. Clin Cancer Res. 2020;26(8):1846–55. 31757877 10.1158/1078-0432.CCR-19-2226

[14] Fenerty KE , Folio LR , Patronas NJ , Marté JL , Gulley JL , Heery CR . Predicting clinical outcomes in chordoma patients receiving immunotherapy: a comparison between volumetric segmentation and RECIST. BMC Cancer. 2016;16(1):1–9. 10.1186/s12885-016-2699-x PMC4995658 27553491

[15] Ferrara R , Mezquita L , Texier M , Lahmar J , Audigier‐Valette C , Tessonnier L , et al. Hyperprogressive disease in patients with advanced non–small cell lung cancer treated with PD‐1/PD‐L1 inhibitors or with single‐agent chemotherapy. JAMA Oncol. 2018;4(11):1543–52. 30193240 10.1001/jamaoncol.2018.3676 PMC6248085

[16] Tunali I , Gray JE , Qi J , Abdalah M , Jeong DK , Guvenis A , et al. Novel clinical and radiomic predictors of rapid disease progression phenotypes among lung cancer patients treated with immunotherapy: an early report. Lung Cancer. 2019;129:75–9. 30797495 10.1016/j.lungcan.2019.01.010 PMC6450086

[17] Ruiz‐Patiño A , Arrieta O , Cardona AF , Martín C , Raez LE , Zatarain‐Barrón ZL , et al. Immunotherapy at any line of treatment improves survival in patients with advanced metastatic non‐small cell lung cancer (NSCLC) compared with chemotherapy (Quijote‐CLICaP). Thorac Cancer. 2020;11(2):353–61. 31828967 10.1111/1759-7714.13272 PMC6996989

[18] Choi YJ , Kim T , Kim EY , Lee SH , Kwon DS , Chang YS . Prediction model for hyperprogressive disease in non‐small cell lung cancer treated with immune checkpoint inhibitors. Thorac cancer. 2020;11(10):2793–803. 32779394 10.1111/1759-7714.13594 PMC7529559

[19] Kato S , Goodman A , Walavalkar V , Barkauskas DA , Sharabi A , Kurzrock R . Hyperprogressors after immunotherapy: analysis of genomic alterations associated with accelerated growth rate. Clin Cancer Res. 2017;23(15):4242–50. 28351930 10.1158/1078-0432.CCR-16-3133 PMC5647162

[20] Marten K , Auer F , Schmidt S , Kohl G , Rummeny EJ , Engelke C . Inadequacy of manual measurements compared to automated CT volumetry in assessment of treatment response of pulmonary metastases using RECIST criteria. Eur Radiol. 2006;16(4):781–90. 16331462 10.1007/s00330-005-0036-x

[21] Wulff A , Fabel M , Freitag‐Wolf S , Tepper M , Knabe H , Schäfer J , et al. Volumetric response classification in metastatic solid tumors on MSCT: initial results in a whole‐body setting. Eur J Radiol. 2013;82(10):e567–e73. 23827800 10.1016/j.ejrad.2013.05.030

[22] Frenette A , Morrell J , Bjella K , Fogarty E , Beal J , Chaudhary V . Do diametric measurements provide sufficient and reliable tumor assessment? An evaluation of diametric, areametric, and volumetric variability of lung lesion measurements on computerized tomography scans. J Oncol. 2015;2015:632943. 26064117 10.1155/2015/632943 PMC4441994

[23] Han D , Heuvelmans MA , Vliegenthart R , Rook M , Dorrius MD , De Jonge GJ , et al. Influence of lung nodule margin on volume‐and diameter‐based reader variability in CT lung cancer screening. Br J Radiol. 2018;91(1090):20170405. 28972803 10.1259/bjr.20170405 PMC6350488

[24] Greenberg V , Lazarev I , Frank Y , Dudnik J , Ariad S , Shelef I . Semi‐automatic volumetric measurement of response to chemotherapy in lung cancer patients: how wrong are we using RECIST? Lung Cancer. 2017;108:90–5. 28625656 10.1016/j.lungcan.2017.02.017

[25] Chen Y , Hu J , Bu F , Zhang H , Fei K , Zhang P . Clinical characteristics of hyperprogressive disease in NSCLC after treatment with immune checkpoint inhibitor: a systematic review and meta‐analysis. BMC Cancer. 2020;20(1):1–9. 10.1186/s12885-020-07206-4 PMC7392646 32727409

[26] Russo A , Russano M , Franchina T , Migliorino MR , Aprile G , Mansueto G , et al. Neutrophil‐to‐lymphocyte ratio (NLR), platelet‐to‐lymphocyte ratio (PLR), and outcomes with nivolumab in pretreated non‐small cell lung cancer (NSCLC): a large retrospective multicenter study. Adv Ther. 2020;37(3):1145–55. 32002809 10.1007/s12325-020-01229-w

[27] Nakaya A , Kurata T , Yoshioka H , Takeyasu Y , Niki M , Kibata K , et al. Neutrophil‐to‐lymphocyte ratio as an early marker of outcomes in patients with advanced non‐small‐cell lung cancer treated with nivolumab. Int J Clin Oncol. 2018;23(4):634–40. 29442281 10.1007/s10147-018-1250-2 PMC6097082

[28] Kiriu T , Yamamoto M , Nagano T , Hazama D , Sekiya R , Katsurada M , et al. The time‐series behavior of neutrophil‐to‐lymphocyte ratio is useful as a predictive marker in non‐small cell lung cancer. PLoS One. 2018;13(2):e0193018. 29447258 10.1371/journal.pone.0193018 PMC5814002

[29] Kosmidis P , Krzakowski M , Investigators E . Anemia profiles in patients with lung cancer: what have we learned from the European cancer Anaemia survey (ECAS)? Lung Cancer. 2005;50(3):401–12. 16191450 10.1016/j.lungcan.2005.08.004

[30] Zhang Z , Zhang F , Yuan F , Li Y , Ma J , Ou Q , et al. Pretreatment hemoglobin level as a predictor to evaluate the efficacy of immune checkpoint inhibitors in patients with advanced non‐small cell lung cancer. Ther Adv Med Oncol. 2020;12:1758835920970049. 33224276 10.1177/1758835920970049 PMC7649885

[31] Suárez C , Morales‐Barrera R , Garcia‐Ruiz A , Gonzalez M , Ligero M , Valverde C , et al. Hyperprogressive disease in patients with metastatic genitourinary tumors treated with immune checkpoint inhibitors. J Clin Oncol. 2019;37(7_suppl):448.30625041

[32] Zhang L , Wu L , Chen Q , Zhang B , Liu J , Liu S , et al. Predicting hyperprogressive disease in patients with advanced hepatocellular carcinoma treated with anti‐programmed cell death 1 therapy. eClinicalMedicine. 2021;31:100673. 33554079 10.1016/j.eclinm.2020.100673 PMC7846667

[33] Seymour L , Bogaerts J , Perrone A , Ford R , Schwartz LH , Mandrekar S , et al. iRECIST: guidelines for response criteria for use in trials testing immunotherapeutics. Lancet Oncol. 2017;18(3):e143–e52. 28271869 10.1016/S1470-2045(17)30074-8 PMC5648544

