# Supplementary material for: Clinical outcomes of hyperprogression based on volumetry in non‐small cell lung cancer after immune checkpoint inhibitor treatment
Source: Thorac Cancer. 2022 Jul 3;13(15):2170–9. doi: 10.1111/1759-7714.14539 (PMC9346184; doi:10.1111/1759-7714.14539)
Supplement: Supplementary file 1 — Table S1 Supporting Information [file TCA-13-2170-s001.docx]

**Supplement Table 1**. Clinical characteristics by hyperprogression using the diametric measurement

|  | All patients  (n=219) | Non-HPDd  (n=180) | HPDd  (n=39) | *P*-value |
| --- | --- | --- | --- | --- |
| **Mean Age** | 65.0 ± 9.0 | 65.1 ± 9.1 | 64.5 ± 8.6 | 0.91 |
| <60 | 63 (28.8%) | 51 (28.3%) | 12 (30.8%) |  |
| ≥60 | 156 (71.2%) | 129 (71.7%) | 27 (69.2%) |  |
| **Gender, (%)** |  |  |  | 0.5 |
| Male | 169 (77.2%) | 141 (78.3%) | 28 (71.8%) |  |
| Female | 50 (22.8%) | 39 (21.7%) | 11 (28.2%) |  |
| **Smoking history, (%)** |  |  |  | 0.33 |
| Ever smoker | 157 (71.7%) | 132 (73.3%) | 25 (64.1%) |  |
| Never smoker | 62 (28.3%) | 48 (26.7%) | 14 (35.9%) |  |
| **ECOG PS, (%)** |  |  |  |  |
| ≤1 | 182 (83.1%) | 148 (82.2%) | 34 (87.2%) | 0.6 |
| >2 | 37 (16.9%) | 32 (17.8%) | 5 (12.8%) |  |
| **Previous Operative therapy, (%)** |  |  |  | 0.86 |
| No | 190 (86.8%) | 157 (87.2%) | 33 (84.6%) |  |
| Yes | 29 (13.2%) | 23 (12.8%) | 6 (15.4%) |  |
| **Previous radiation therapy, (%)** |  |  |  |  |
| No | 133 (60.7%) | 108 (60.0%) | 25 (64.1%) | 0.76 |
| Yes | 86 (39.3%) | 72 (40.0%) | 14 (35.9%) |  |
| **EGFR mutation, (%)** |  |  |  | 0.38 |
| No | 20 (9.1%) | 5 (12.8%) | 15 (8.3%) |  |
| Yes | 199 (90.9%) | 34 (87.2%) | 165 (91.7%) |  |
| **Tumor pathology, (%)** |  |  |  | 1.00 |
| Non SQC | 77 (35.2%) | 63 (35.0%) | 14 (35.9%) |  |
| SQC | 142 (64.8%) | 117 (65.0%) | 25 (64.8%) |  |
| **PD-L1 expression status, (%)** |  |  |  | 0.24 |
| <50% | 68 (33.0%) | 53 (31.0%) | 15 (42.9%) |  |
| ≥50 | 138 (67.0%) | 118 (69.0%) | 20 (57.1%) |  |
| **Bone metastasis** |  |  |  | 0.34 |
| No | 146 (66.7%) | 123 (68.3%) | 23 (509.0%) |  |
| Yes | 73 (33.3%) | 57 (31.7%) | 16 (41.0%) |  |
| **Brain metastasis** |  |  |  | 1.0 |
| No | 179 (81.7%) | 148 (82.2%) | 31 (79.5%) |  |
| Yes | 40 (18.3%) | 32 (17.8%) | 8 (20.5%) |  |
| **Liver metastasis** |  |  |  | 0.48 |
| No | 195 (89.0%) | 162 (90.0%) | 33 (84.6%) |  |
| Yes | 24 (11.0%) | 18 (10.0%) | 6 (15.4%) |  |
| **Adrenal metastasis** |  |  |  | 0.65 |
| No | 203 (92.7%) | 168 (93.3%) | 35 (89.7%) |  |
| Yes | 16 (7.3%) | 12 (6.7%) | 4 (10.3%) |  |
| **Metastatic site** |  |  |  | <0.01 |
| ≤2 | 186 (84.9%) | 161 (89.4%) | 25 (64.1%) |  |
| >2 | 33 (15.1%) | 19 (10.6%) | 14 (35.9%) |  |
| **Treatment lines before ICI** |  |  |  | 0.66 |
| 1 or 2 | 182 (83.1%) | 151 (83.9%) | 31 (79.5%) |  |
| 3 or more | 37 (16.9%) | 29 (16.1%) | 8 (20.5%) |  |
| **Type of ICI** |  |  |  | 0.31 |
| Nivolumab | 121 (55.3%) | 95 (52.8%) | 26 (66.7%) |  |
| Pembrolizumab | 84 (38.4%) | 74 (41.1%) | 10 (25.6%) |  |
| Atezolizumab | 13 (5.9%) | 10 (5.6%) | 3 (7.7%) |  |
| Durvalumab | 1 (0.5%) | 1 (0.6%) | 0 (0.0%) |  |
| **Mean NLR** | 3.7 ± 3.0 | 3.6 ± 2.8 | 4.4 ± 3.4 | 0.01 |
| <3.3 | 127 (58.0%) | 112 (62.2%) | 15 (38.5%) |  |
| ≥3.3 | 92 (42.0%) | 68 (37.8%) | 24 (61.5%) |  |
| **Mean PLR** | 196.8 ± 112.4 | 190.8 ± 106.0 | 212.7 ± 135.9 | 0.34 |
| <214 | 141 (64.4%) | 119 (66.1%) | 22 (56.4%) |  |
| ≥214 | 78 (35.6%) | 61 (33.9%) | 17 (43.6%) |  |
| **Hemoglobin level, g/dL** | 11.6 ± 1.8 | 11.0 ± 1.8 | 11.0 ± 1.7 | 0.05 |
| <10 | 45 (20.5%) | 32 (12.8%) | 13 (33.3%) |  |
| ≥10 | 174 (79.5%) | 148 (82.2%) | 26 (66.7%) |  |
| **Albumin level, g/dL** | 3.9 ± 0.5 | 3.9 ± 0.5 | 3.7 ± 0.5 | 0.33 |
| <3.5 | 46 (21.2%) | 35 (19.7%) | 11 (28.2%) |  |
| ≥3.5 | 171 (78.8%) | 143 (80.3%) | 28 (71.8%) |  |
| **LDH, U/L** | 388.6 ± 255.4 | 373.1 ± 254.2 | 455.3 ± 254.4 | 0.51 |
| <450 | 111 (72.1%) | 92 (73.6%) | 19 (65.5%) |  |
| ≥450 | 43 (27.9%) | 33 (26.4%) | 10 (34.5%) |  |

HPDv, patients with HPD assessed by volumetric method; ECOG PS, Eastern Cooperative Oncology Group performance status; SQC, squamous cell carcinoma; ICI, Immune-checkpoint inhibitors; NLR, neutrophil-lymphocyte ratio; PLR, platelet-lymphocyte ratio;
